# Performance of Contrast-Enhanced Mammography (CEM) for Monitoring Neoadjuvant Chemotherapy Response among Different Breast Cancer Subtypes

**DOI:** 10.3390/cancers16152694

**Published:** 2024-07-29

**Authors:** Sofia Vidali, Giovanni Irmici, Catherine Depretto, Chiara Bellini, Francesca Pugliese, Ludovica Anna Incardona, Federica Di Naro, Diego De Benedetto, Giacomo Di Filippo, Fabiola Ferraro, Claudia De Berardinis, Vittorio Miele, Gianfranco Scaperrotta, Jacopo Nori Cucchiari

**Affiliations:** 1 Breast Imaging Unit, Department of Radiology, Careggi University Hospital, 50134 Florence, Italy; bellinich@aou-careggi.toscana.it (C.B.); ludovicaanna.incardona@unifi.it (L.A.I.); debenedettod@aou-careggi.toscana.it (D.D.B.);; 2 Institute of Clinical Physiology, National Research Council, 56124 Pisa, Italy; 3 Breast Radiology, Fondazione IRCCS Istituto Nazionale dei Tumori, 20133 Milan, Italy; 4 UOC Endocrinochirurgia, Azienda Ospedaliera Universitaria Integrata Verona, 37134 Verona, Italy; giacomo.difilippo@aovr.veneto.it; 5 Department of Biomedicine Neuroscience and Advanced Diagnostics (BiND), University of Palermo, 90133 Palermo, Italy; 6 Department of Radiology, Careggi University Hospital, 50134 Florence, Italy

**Keywords:** contrast-enhanced mammography, CEM, neoadjuvant therapy, locally advanced breast cancer, pCR, breast cancer biologic features

## Abstract

**Simple Summary:**

The indications for neoadjuvant chemotherapy (NAT) have expanded in recent years both for locally advanced and early-stage breast cancer, with different pathological complete response (pCR) rates among different molecular subtypes. This retrospective two-center study aims to evaluate the diagnostic performance of Contrast-Enhanced Mammography (CEM) in assessing the response to NAT in breast cancer patients. The CEM sensitivity and specificity were 66.2% and 75.2%, with the highest specificity (80.9%) in HR+/HER2− and the highest sensitivity (70%) in triple-negative breast cancer. CEM is a valid tool to assess the pCR with different performances among the molecular subtypes and may be reliable in the decisional process of de-escalating surgical management.

**Abstract:**

Neoadjuvant chemotherapy (NAT) plays a crucial role in breast cancer (BC) treatment, both in advanced BC and in early-stage BC, with different rates of pathological complete response (pCR) among the different BC molecular subtypes. Imaging monitoring is mandatory to evaluate the NAT efficacy. This study evaluates the diagnostic performance of Contrast-Enhanced Mammography (CEM) in BC patients undergoing NAT. This retrospective two-center study included 174 patients. The breast lesions were classified based on the molecular subtypes in hormone receptor (HR+)/HER2−, HER2+, and triple-negative breast cancer (TNBC). The histopathological analysis performed following surgery was used as a reference standard for the pCR. Sensitivity, specificity, PPV, and NPV were measured overall and for the different subtypes. We enrolled 174 patients, 79/174 (46%) HR+/HER2−, 59/174 (33.9%) HER2+, and 35/174 (20.1%) TNBC; the pCR was found in 64/174 (36.8%), of which 57.1% were TNBCs. In the total population, the CEM sensitivity and specificity were 66.2% and 75.2%, with a PPV of 61.4% and an NPV of 78.8%. The highest specificity (80.9%) and NPV (91.7%) were found in HR+/HER2−, while the highest sensitivity (70%) and PPV appeared (73.7%) in TNBC. The results indicate that CEM is a valid tool to assess the pCR, with different performances among the subtypes of BC.

## 1. Introduction

Neoadjuvant chemotherapy (NAT) plays a crucial role in breast cancer (BC) treatment. NAT is indicated in locally advanced breast cancer to reduce the tumor size, thus facilitating breast-conserving surgery [1,2]. NAT enables the early assessment of the therapeutic response, which aids in optimizing the subsequent treatment plans and enhancing the overall treatment efficacy. For this reason, NAT indication has also been extended more recently to early-stage breast cancer [1,3]. The response to NAT is particularly influenced by the molecular BC subtypes, resulting in higher pathological complete response (pCR) rates in triple-negative breast cancer (TNBC) and HER2+ [4,5,6]. This has prompted recent studies to evaluate the feasibility of omitting surgery in patients who achieve a radiological complete response (rCR) to NAT [7,8]. Therefore, accurate imaging monitoring of the treatment response is crucial to optimize the therapeutic strategies and improve the patient outcomes [9], in particular with functional diagnostic techniques such as MRI and Contrast-Enhanced Mammography (CEM) [9]. MRI is recognized as the gold standard [10,11]; however, CEM can be considered an alternative, especially in the case of MRI contraindication [12,13]. This study aims to explore the diagnostic performance of CEM in BC patients undergoing NAT, overall and among different BC molecular subtypes.

## 2. Materials and Methods

This retrospective study was conducted in two tertiary-level cancer centers between January 2016 and May 2024. We enrolled consecutive adult patients referred to the breast radiology department for invasive breast cancers treated with NAT followed by surgery. Exclusion criteria included male sex, history of adverse reaction to iodinate contrast agent, and kidney function impairment. NAT modified or not entirely carried out, no availability of post-NAT CEM. The NAT regimen was established by medical oncologists according to guidelines, considering the molecular features of the lesions.

### 2.1. CEM Examination

The CEM examinations were performed with Selenia™ Dimensions™ mammography system (Hologic, Marlborough, MA, USA) and Fujifilm Amulet innovality mammography system (Fujifilm, Akasaka Minato-ku, Tokyo, Japan). The contrast medium used was Xenetix 350 mg/mL or Iopamire 370 mg/mL, 1.5 mg/kg bodyweight (maximum 100 mg). It was administered by a peripheral venous access in the arm. After 2 min from the s of injection, compression was started. Dual imaging with high- and low-energy images at each projection was performed following this order: cranio-caudal (CC) projection and mediolateral-oblique (MLO) projection of the affected side, and CC projection and MLO projection of the unaffected side. After 7 min, the four projections were acquired again in the same order for the delayed phase. The total acquisition time was between 10 and 12 min. Patients were observed after the contrast media injection to discover potential allergic reactions.

### 2.2. Image Analysis

**Figure 1:**
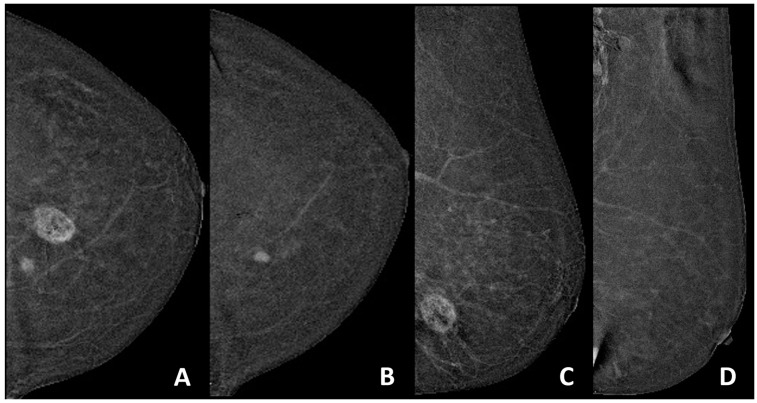
HER2+ disease in the left breast showing pCR following NAC with only a residual enhancement visible in CC projection in the mid-inner quadrant corresponding to a known charcoal granuloma. (**A**,**C**) Pre-NAT recombined early CC and MLO CEM projections of the left breast. (**B**,**D**) Post-NAT recombined early CC and MLO CEM projections of the left breast.

Two radiologists with experience in breast imaging and expertise in CEM (R1 from center A with 15 years of experience in breast radiology and at least 8 years of experience in CEM and R2 form center B with 11 years of experience in breast radiology and at least 6 years of experience in CEM) reviewed the examinations of their centers. Readers were blinded to the clinical and histopathological information. rCR to NAT was defined as the absence of enhancement comparing post-NAT with pre-NAT CEM in accordance with RECIST version 1.1 and reported using CEM BI-RADS lexicon [10,11]. An example of a case considered as rCR is presented in Figure 1.

**Figure 2:**
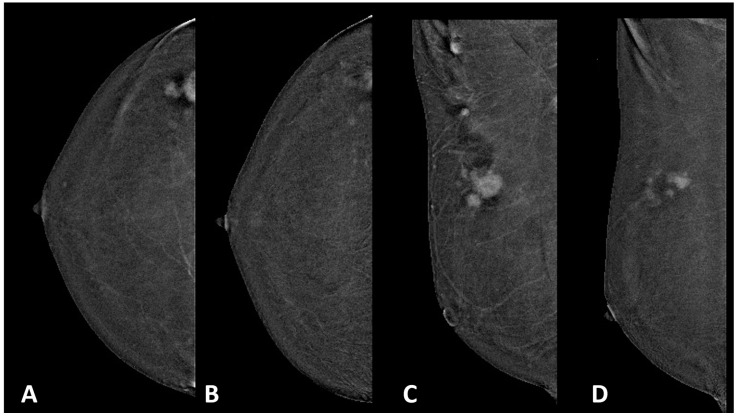
HR+/HER2− cancer in the superior outer quadrant of the right breast showing partial response after NAT. (**A**,**C**) Pre-NAT recombined early CC and MLO CEM projections of the right breast. (**B**,**D**) Post-NAT recombined early CC and MLO CEM projections.

Before the study, the two radiologists reviewed 20 cases simultaneously to standardize the readings: the two radiologists produced a report including the following information: rCR, residual disease, and extension of residual disease. Then, a meeting between the two was organized to exchange imaging and produce additional reports with same descriptors on the counterpart selected exams. Discrepancies between the readers were then discussed between the two, and, in case of no resolution, one additional breast radiologist with the same experience from the hosting center was consulted to solve it. An example of a partial response with the agreement of the two readers is presented in Figure 2.

### 2.3. Pathologic Review

All patients included in the study underwent 14 G ultrasound-guided core needle biopsy or 8–9 G stereotactic or tomo-guided biopsy before NAT. Lesions were divided into three groups according to molecular classification.

- Hormone receptor (HR)+/HER2−: ER and/or PR positivity (at least 1% of the cancer cells) and HER2 0/1+ at immunohistochemistry.
- HER2+ HR−: ER and PR negativity and HER2 3+ or 2+ along with HER2 gene amplification by fluorescence in situ hybridization on core biopsy.
- Triple-negative breast cancer (TNBC): ER and PR negativity and HER2 0 or 1+.
- PCR was defined as absence of invasive cells in surgical specimen (ypT_0~Tis_).
- Ki67 levels were classified as low if ≤20% and high if >20% [14].

### 2.4. Statistical Analysis

Continuous variables were defined as median and interquartile range (IQR). Categorical variables were expressed as absolute values and relative frequencies. Differences between groups were tested with chi-squared test for dichotomous variables and Mann–Whitney test for continuous variables, respectively. CEM performance in diagnosing pCR was measured by calculating sensitivity, specificity, positive predictive value (PPV), and negative predictive value (NPV) in the whole population and for each breast cancer molecular subtype. True-positives were defined when imaging showed no enhancement and histology showed pCR, true-negative when imaging showed enhancement and histology residual disease. A multivariate logistic regression analysis with CEM rCR as a variable correcting for confounding factors, specifically molecular subtypes, patients’ age, and lesions dimensions, was performed. Statistical analysis was performed with SPSS v 25 (IBM Corp., Armonk, NY, USA). *p* < 0.05 was considered statistically significant.

## 3. Results

We enrolled 174 patients (median age 56 years; IQR 50–67 years; *p* 0.350), and the overall median lesion size was 25 mm (IQR 17–30 mm; *p* 0.214). At biopsy, 79/174 (46%) were HR+/HER2−, 59/174 (33.9%) were HER2+, and 35/174 (20.1%) were TNBC.

After NAC, 70/174 CEM performances (40.2%) were classified as rCRs. Specifically, TNBC and HER2+ reached a similar ratio with 19/35 (54.3%) and 32/59 (54.2%), respectively, followed by 19/80 (23.8%) HR+/HER2. The population features related to rCR are reported in Table 1.

Regarding the surgical specimens, a pCR was found in 64/174 (36.8%) patients, with the highest proportion of them being TNBC, 20/35 (57.1%), followed by 33/59 (55.9%) HER2+ and 11/79 (13.8%) HR+/HER2.

Upon the multivariate analysis, no statistically significant correlation was found between the rCR and age or lesions’ dimensions and ki-67 (*p* = 0.226, *p* = 0.301 and *p* = 0.225), while the molecular subtype correlated significantly with the rCR (*p* < 0.001).

In terms of the diagnostic performance in the global population, the sensitivity and specificity of CEM were 66.2% (95% CI; 54.2–76.9%) and 75.2% (66.6–82.7%), respectively, with a PPV of 61.4% (95% CI; 49.8–72.2%) and NPV of 78.8% (70.4–85.9%).

Upon the post hoc analysis, however, considering the pCR patients with a residual DCIS as non-pCR, the sensitivity increased to 67.3% (95% CI; 54.3–78.8%).

The diagnostic performance of CEM exhibited differences among the various subtypes:

- For HR+/HER2−, the sensitivity, specificity, PPV, and NPV were, respectively, 54.5% (95% CI; 26.5–80.6%), 80.9% (70.5–89%), 31.6% (95% CI; 53.9–87.7%), and 91.7% (82.9–96.9%);
- For HER2+, the sensitivity, specificity, PPV, and NPV for HER2+ were, respectively, 69.7% (95% CI; 53–83.5%), 65.4% (46.3–81.6%), 71.9% (95% CI; 55–85.4%), and 63% (44.2–79.4%);
- For TNBC, the sensitivity, specificity, PPV, and NPV were, respectively, 70% (95% CI; 48.3–86.8%), 66.7% (95% CI; 41.5–86.5%), 73.7% (95% CI; 51.7–89.7%), and 62.5% (95% CI; 38.2–83.0%).

The diagnostic performance is resumed in Table 2.

**Figure 3:**
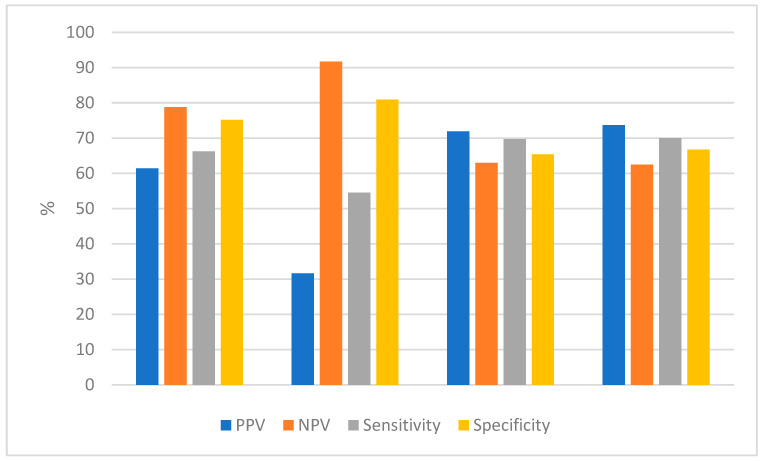
Test performance in terms of PPV, NPV, sensitivity, and specificity in the general population and each molecular subgroup.

The PPV, NPV, sensitivity, and specificity of each molecular subgroup and of the general population are reported in Table 3 and Figure 3.

The PPV, NPV, sensitivity, and specificity of each molecular subgroup and of the general population are reported in Table 3. No significant correlation was found between Ki-67 and rCR (*p* 0.174).

## 4. Discussion

This retrospective two-center study evaluated the diagnostic performance of CEM in the assessment of the tumor response to NAT in patients with BC, overall and among the different molecular subtypes. Monitoring the response to NAT is critical for determining additional treatments, particularly in terms of surgical management with a future view to omitting surgery in exceptional-responder patients [12,13]. In this context, CEM has proven to be a valid alternative to MRI in assessing the response after NAT [6]; to date, this is one of the studies with the largest sample size. The overall sensitivity of our sample was 66.2%, and the specificity was 75.2%. Bernardi et al., in a prospective study including 51 patients, reported a sensitivity and specificity of CEM in predicting the pCRs of 81% and 83%, respectively [13]. Hogan et al. reported similar data in terms of sensitivity (81%) with lower specificity (50%) [15], while Kaiyin et al. in a recent meta-analysis of six studies with 328 patients reported pooled sensitivity and specificity for CEM of 93% and 68%, respectively [16]. In the systematic review by Lobbes et al., the accuracy of MRI in detecting the pCR in seven studies had median sensitivity and specificity values of 42% and 89%, with PPV and NPV values of 64% and 87%, respectively [10]. In the meta-analysis by Yuan et al., the pooled weighted sensitivity and specificity of 25 articles examining MRI for the pCR reached 63% and 91%, respectively [17]. In the meta-analysis by Tang et al. including 24 papers on the performance of either CEM, MRI, or both after NAT, the pooled sensitivity and specificity for MRI resulted in 77% (95% CI; 67–0.84%) and 82% (95% CI; 73–89%), while for CEM 83% (95% CI; 66–93%) and 82% (95% CI; 68–91%) [18]. However, the specificity and sensitivity demonstrated in our findings are at the lower end of the range that has been previously shown. This may be attributed to several factors, including differences in the study populations, variations in the mammographic techniques among the centers, and the different NAT protocols. Due to variations in the molecular distribution of the patient population, the pre-test probability of achieving the pCR can vary widely. Future studies taking into account these molecular differences by recruiting balanced populations and employing uniform treatment strategies may allow us to better define the predicting power of CEM. Additionally, the lack of standard CEM or imaging interpretation protocols may explain the discrepancy between our findings and others. When analyzing our findings by molecular subtype, the CEM sensitivity was higher for HER2+ and TNBC (69.7% and 70%) compared to the HR+/HER2− forms (54.5%), while the specificity was higher for the HR+/HER2− forms (80.9%) compared to the other subgroups (65.4% and 66.7%). These data can be explained by the influence of the molecular phenotype on the performance of functional imaging, which is related to higher proliferation rates and increased angiogenesis in TNBC and HER2+ tumors compared to the HR+/HER2− types [19,20,21]. As shown in the study by Negrao et al. and McGuire et al. on the role of MRI in predicting the pCR after NAT, the sensitivity was lower for the HR+/HER2− subtypes, and the molecular subtype was found to be related to the MRI accuracy in predicting the pCR [22,23]. More specifically, a negative relationship between HR+ and pCR has been previously demonstrated. In the meta-analysis by Houssami et al., the odds of a pCR were the highest for the HER2+/TNBC HR− subgroups [24], while, in the meta-analysis by Wu et al., the pCR rates in those patients with TNBCs were significantly higher than in non-TNBC ones [25]. Therefore, defining the tumor molecular subtype prior to NAT is crucial in determining patients who are likely to exhibit an effective treatment response. Additional attention is to be paid to the PPV and NPV of CEM in the different molecular subgroups. CEM showed a good PPV both for HER2+ and TNBC compared to the HR+/HER2− subtypes and a high NPV for the HR+ variants. This can be interpreted as a high likelihood of a pCR when those patients with aggressive tumor subtypes meet the imaging criteria for an rCR. This is especially true for TNBC, whereas, in the absence of an rCR, a pCR is most probably not achievable both in the HR+/HER2− variants and also, although with weaker strength, in HER2+ and TNBC. Similar results were obtained in the study by Janssen et al. on CE-MRI, where the PPV for the pCR was the highest in the HR−/HER2− subtype and lowest in the HR+/HER2− subtype [26]. Canteros et al. showed that CEM had higher sensitivity among HR− tumors compared to HR+ (88.9% vs. 63.6%) [21]. However, these results should be interpreted with the pre-test probability of each subtype to reach the pCR in mind. More aggressive subtypes more likely reach the pCR compared to HR+/HER2− ones. In our sample, the PPV for the HR+/HER2− cancers was 31.6%, and the pCR was achieved in 13.8% of this subgroup, meaning that, although the likelihood to have a pCR with an rCR is not very high, the baseline potential of the HR+/HER2− cancer to reach the pCR is very low. This is confirmed by the pCR rates obtained, to be considered as substitutes of the pre-test probabilities to reach the pCR. The latter influences the chances to have a high PPV, giving it a higher relative weight in predicting the pCR. Conversely, the NPV was 91.7% for the HR+/HER2− cancers, with 86.2% residual disease on the post-surgical report (pCR), resulting in the lower relative strength of the predictive value. Similar considerations can be made for the PPV of CEM for the pCR in the HER2+ and TNBC groups. Although the PPV rates are 71.9% and 73.7%, respectively, a pCR was observed in 55.9% and 57.1%, respectively. This can be interpreted as a higher likelihood of achieving a pCR for more aggressive subtypes compared to HR+/HER2− ones, as expected from the prior literature, therefore leading to a higher PPV of the tested technique. The tight correlation between the rCR and pCR demonstrates that CEM is not only a reliable tool in assessing the outcomes after NAT but also a valid medium to translate the intrinsic characteristics, like vascularization and aggressiveness, and the biologic behaviors of the different molecular subtypes into measurable visual parameters. In our study, the Ki67 levels did not show a significant correlation with the CEM rCR after NAT. Similar results were observed in the work of Luczynska et al. on 145 lesions, where the absolute enhancement value of CEM was similar for Ki67-low and Ki67-high tumors [27]. This may be due to multiple factors, including the sample heterogeneity and an unbalanced distribution of the subtypes as well as the lesions’ dimensions. This study should be interpreted within the context of its design. First, we did not assess the influence of the lesion enhancement morphology, conspicuity, kinetics, and presence of background parenchymal enhancement (BPE) on the CEM performance in detecting the pCR. Additionally, second-look modalities such as mammography and ultrasonography were not included. There may have also been inconsistencies in the machine performance and technical approach to imaging acquisition between the two centers that participated. Another possible area of variability lies in the chemotherapeutic regimens. High false-negative rates in HER2− patients were reported by Chen al, particularly when treated with anti-angiogenic drugs [28]. The inclusion of DCIS in the pCR group requires further investigation. The CEM accuracy if DCIS is included in the definition of the pCR is to be considered since DCIS should also be excised and should definitely be identified in the perspective of de-escalating surgical treatments [29,30]. In the future, we plan to include more centers to improve the generalizability of our findings.

## 5. Conclusions

Overall, CEM is accurate in assessing the pCR and predicting the pathologic-complete response among the different molecular subtypes after NAT. However, multiple factors, such as the treatment protocols, heterogeneity of the distribution of biological features, as well as CEM-related features such as BPE and enhancement morphology, may influence the test performance and should be further characterized in wider multi-center studies to establish the role of CEM clinical decision-making. These future considerations are especially important to determine the safety of de-escalating surgical intervention for selected rCR patients.

## Figures and Tables

**Table 1:** General population characteristics based on rCR.

General Population		rCR						*p*
		No			Yes			
		Median	Count	N%	Median	Count	N%	
Age (y.o.)		55			58			
Dimension (mm)		27			25			
rCR	no					70	100%	
	yes		104	100%				
pCR	no		82	78.8%		27	38.6%	0.000
	yes		22	21.2%		43	61.4%	
HR+/HER2−	no		44	42.3%		51	72.9%	0.000
	yes		61	57.7%		19	27.1%	
HER2+	no		77	74.0%		38	54.3%	0.007
	yes		27	26.0%		32	45.7%	
TNBC	no		88	84.6%		51	72.9%	0.058
	yes		16	15.4%		19	27.1%	
Ki67 > 20%	no		32	30.1%		15	21.5%	0.225
	yes		72	69.9%		55	78.5%	

**Table 2:** rCR and pCR with % in each molecular subgroup.

CR in Molecular Subgroups		HR+/HER2−			HER2+			TNBC		
		pCR (%)			pCR (%)			pCR (%)		
		No	Yes	Tot	No	Yes	Tot	No	Yes	Tot
rCR (%)	no	55 (69.6%)	5 (6.3%)	61 (76.2%)	17 (28.8%)	10 (16.9%)	27 (45.8%)	10 (28.6%)	6 (17.1%)	16 (45.7%)
	yes	13 (16.5%)	6 (7.6%)	19 (23.8%)	9 (15.3%)	23 (39.0%)	32 (54.2%)	5 (14.3%)	14 (40.0%)	19 (54.3%)
	tot	69 (86.2%)	11 (13.8%)	80 (100%)	26 (44.1%)	33 (55.9%)	59 (100%)	15 (42.9%)	20 (57.1%)	35 (100%)

**Table 3:** Test performance in terms of PPV, NPV, sensitivity, and specificity in the general population and each molecular subgroup.

Population Groups		General	HR+/HER2−	HER2+	TNBC
PPV	%	61.4	31.6	71.9	73.7
	95% CI	49.8–72.3	53.9–87.69	55.0–85.4	51.7–89.7
NPV	%	78.8	91.7	63.0	62.5
	95% CI	70.4–85.9	82.9–96.9	44.2–79.4	38.2–83.0
Sens	%	66.2	54.5	69.7	70.0
	95% CI	54.2–76.9	26.5–80.6	53.0–83.5	48.3–86.8
Spec	%	75.2	80.9	65.4	66.7
	95% CI	41.3–66.6	70.5–89.0	46.3–81.6	41.5–86.5

## Data Availability

The raw data supporting the conclusions of this article will b made available by the authors on request.
